# Endocrine Disrupting Chemicals and Endometrial Cancer: An Overview of Recent Laboratory Evidence and Epidemiological Studies

**DOI:** 10.3390/ijerph14030334

**Published:** 2017-03-22

**Authors:** Maddalena Mallozzi, Chiara Leone, Francesca Manurita, Filippo Bellati, Donatella Caserta

**Affiliations:** Surgical and Medical Department of Translational Medicine, Sant’Andrea Hospital, Faculty of Medicine and Psycology, University of Rome “Sapienza”, Via di Grottarossa n° 1035, 00139 Rome, Italy; maddalenamallozzi@gmail.com (M.M.); chiara.leone1991@gmail.com (C.L.); francesca.manurita@uniroma1.it (F.M.); filippo.bellati@uniroma1.it (F.B.)

**Keywords:** endocrine disruptors, endometrial cancer, bisphenol A, polychlorinated biphenyls, dioxin, cadmium

## Abstract

*Background*: Although exposure to endocrine disruptor compounds (EDCs) has been suggested as a contributing factor to a range of women’s health disorders including infertility, polycystic ovaries and the early onset of puberty, considerable challenges remain in attributing cause and effect on gynaecological cancer. Until recently, there were relatively few epidemiological studies examining the relationship between EDCs and endometrial cancer, however, in the last years the number of these studies has increased. *Methods*: A systematic MEDLINE (PubMed) search was performed and relevant articles published in the last 23 years (from 1992 to 2016) were selected. *Results*: Human studies and animal experiments are confirming a carcinogenic effect due to the EDC exposure and its carcinogenesis process result to be complex, multifactorial and long standing, thus, it is extremely difficult to obtain the epidemiological proof of a carcinogenic effect of EDCs for the high number of confusing factors. *Conclusions*: The carcinogenic effects of endocrine disruptors are plausible, although additional studies are needed to clarify their mechanisms and responsible entities. Neverthless, to reduce endocrine disruptors (ED) exposure is mandatory to implement necessary measures to limit exposure, particularly during those periods of life most vulnerable to the impact of oncogenic environmental causes, such as embryonic period and puberty.

## 1. Introduction

According to the definition of the World Health Organization (WHO), an endocrine disrupting chemical (EDC) is an exogenous substance or mixture that alters the function(s) of the endocrine system and consequently causes adverse effects in an intact organism, or its progeny, or (sub)populations [1]. EDCs are a heterogeneous group of chemicals, comprising persistent contaminants (e.g., dioxins, polychlorinated biphenyl and brominated flame retardants), pesticides (e.g., triazoles, dicarboximides and triazines), industrial substances (e.g., phtalates and bisphenol A) and natural substances (e.g., phytoestrogens).The constant human exposure to environmental and nutritional compounds with endocrine abilities, accentuates the urgency of understanding the effects of these compounds on human health. More and more studies are confirming the complexity and multifactorial effects caused by ED exposure, however, due to the high number of confusing factors, it is difficult to obtain a firm epidemiological demonstration of EDC-related carcinogenesis.

Although exposure to EDCs has been suggested as a contributing factor to a range of women’s health disorders, including infertility [2,3], polycystic ovaries and the early onset of puberty, considerable challenges remain in attributing cause and effect in gynaecological cancer. Malignancies of the female reproductive tract are the fourth most common cancer in women [4] and endometrial cancer accounts for the majority of these cancers. The development of cancer is known to be a multifactorial process and environmental factors can influence and regulate this biological processes through a wide range of actions. Established risk factors for the development of endometrial cancer include high Body Mass Index (BMI) and exposure to estrogens or synthetic estrogenic compounds such as EDCs.

The most significant EDCs are environmental pollutants or major constituents of preservatives and industrial plasticizers and approximately 800 chemicals are known or suspected to have the potential to function as EDCs [5]. This wide range of environmental pollutants, such as polychlorinated biphenyls, dioxins, polycyclic aromatic hydrocarbons, phtalates, bisphenol A and pesticides potentially disrupt hormone metabolic pathways by mimicking the functions of endogenous hormones and in some cases, completely blocking their functions [6].

EDCs are compounds with mostly estrogenic activities and considering that recently, natural estrogens have been classified as known human carcinogens [7] it is plausible that EDCs can be considered oncogenic factors.Estrogens can act as potent mitogens in estrogen receptor (ER)-positive cells with continual exposure to an estrogenic stimulus, having the potential to promote DNA instability, cellular hyperplasia and neoplastic transformation of epithelial cells into carcinomas [8].

As a matter of fact, the steroid hormone 17β-estradiol (E2) is a key regulator of growth, differentiation, and function in a wide array of target tissues, including the male and female reproductive tracts and mammary gland [9]. In addition to the physiological actions of estrogens, inappropriate responses to this hormone have been shown to underlie the pathology of most breast, ovarian and uterine cancers [10].

Originally thought to exert their actions primarily through nuclear hormone receptors, such as estrogen (ERs) and progesterone receptors (PRs), research now clearly demonstrates that the mechanisms of action of EDCs are much broader than originally recognized, including also epigenomic changes [11].

Although several environmental compounds exert their carcinogenic action inducing genetic mutations [12,13], the vast majority of endocrine disruptors do not alter DNA sequences. “Environmental epigenetics” is defined as the ability of an environmental factor to directly act and alter epigenetic processes to promote gene expression and phenotype (physiological characteristics) alterations. The altered epigenetic mark(s) at a specific DNA site in response to an environmental factor to influence gene expression is termed an “epimutation” [14]. Therefore, DNA sequence changes are genetic mutations, while environmentally altered epigenetic sites that influence genome activity are epimutations [15].

In the event an environmental toxicant such as an endocrine disruptor modifies the epigenome of a somatic cell, this may promote disease in the exposed individual, but not be transmitted to the next generation. In the event a toxicant modifies the epigenome of the germ line permanently, then the disease promoted can become transgenerationaly transmitted to subsequent progeny [16].

Environmental epigenetics provides direct molecular mechanisms for factors or toxicants to influence the genetic cascade of events involved in the development. Critical windows of susceptibility exist where these factors modify and affect important stages of development. Generally, these critical windows are early in development when the developing organ system are sensitive to alterations in the epigenome. Modification of the epigenome may continue throughout development, subsequently affecting the adult transcriptome and making tissue, such as the breast, susceptible to developing disease. Importantly, in mammals exposure of gestating females to environmental factors or toxicants during the period of gonadal sex determination can alter epigenetic transgenerational inheritance. The epigenetic reprogramming and imprinting of germ cells can thus allow transgenerational transmission of adult-onset disease phenotypes [17].

A systematic MEDLINE (PubMed) search was performed using the following keywords: “endocrine disruptors”, “endometrial cancer”, “bisphenol A”, “polychlorineted biphenyls”, “dioxin” and “cadmium”.Relevant articles published in the last 23 years (from 1992 to 2016) were selected. We considered experimental studies, epidemiological studies and previous reviews. Criteria for inclusion and exclusion were established before the bibliographic search.Studies published in languages other than English were considered only if an abstract was available. Searching for endocrine disruptors and endometrial cancer only 15 studies were found. Two were excluded because they regarded only the effects of endocrine disruptors on breast cancer genesis and one because it was in the French language. Therefore only 12 studies were determined to be useful (Table 1).

Clinically, endometrial cancer have been divided into estrogen-dependent type I and the less common but clinically more aggressive, estrogen-independent type II. In women, the majority of endometrial cancers are estrogen-dependent type I cancers with increased risk associated with exposure to excess estrogens. It is well established that type I endometrial cancer usually occurs due to unopposed estrogen stimulation [29] and therefore it would be no hard to believe that the estrogenic action of endocrine disruptors can influence its genesis through this pathway as well as through others. In fact, dysregulation of miRNA expression has been implicated in estrogen-related diseases including endometrial cancer.

Estrogens regulate miRNA transcription through estrogen receptors α and β in a tissue-specific and cell-dependent manner and it would lead to the fact that endocrine disruptors could be involved in cancerogenesis also through this mechanism [30]. Nevertheless every category of EDC has a specific mechanism of action that sometime can differ from the others [31]. This is the reason why we decided to identify the different mechanisms of action and correlation with endometrial cancer described for every mostly discussed EDC, not excluding the fact that individuals can be concurrently exposed to different EDCs (cocktail effect) generating impredictable interactions.

## 2. Polychlorinated Biphenyls and Endometrial Cancer

Polychlorinated biphenyls (PCBs) are chemically stable, lipid soluble pollutants. These compounds were used widely in industry for a wide variety of purposes, ranging from plastic resins to carbonless copy paper. Despite their utility, PCBs are linked to environmental and human health risks [32,33] that have resulted in the subsequent banning or highly restricted use of PCBs in many countries. However, it is estimated that >1 million tons of PCBs have been produced, and >70% of the PCBs made are still in use [34,35]. Because of the high stability of PCBs, they persist in the environment and have been detected throughout the food chain [36,37]. Of direct concern to public health is the reported accumulation of PCBs in human tissues, breast milk, and reproductive organs [38,39]. PCBs have been categorized as a Group 2A carcinogen (probably carcinogenic to humans) and they are commonly divided into dioxin-like and non-dioxin like types, based on their similarity or not to tetrachlorodibenzo-*p*-dioxin (TCDD).

Although their adverse impacts on the risk of cancer are of great concern and studies on PCB mixtures have been extensive over the last several decades—including those on endometrial cancer (Table 2), a mechanistic understanding of how PCBs can be implicated, has remained elusive. Their carcinogenic effect would be due to the fact that it can induce potent endothelial cell activation, enhance cellular oxidative stress, and stimulate a variety of potential prometastatic processes, such as increased permeability across the vascular endothelium and induction of adhesion molecules [40,41,42].

PCBs have been proposed to act through a variety of cellular pathways. As previously mentioned, PCBs are suspected to promote cancer through affecting the cellular redox environment.

Regarding endometrial cancer, molecular epidemiological studies have shown how the activity of superoxide dismutase (SOD) would be lower in the endometrial cancer tissue than in the normal endometrium [43,44] and PCBs have been recently showed to activate the enzymatic activity of SOD 1 in endometrial cancer cells [45] as shown in a previous study where PCBs enhanced the activity of SOD both in MCF-10A human breast cells and RWPE-1 human prostate epithelial cells [46].

Of particular interest is the potential estrogenic activity of these compounds that have been demonstrated in vitro and in vivo [47,48,49]. These studies reveal that the estrogenic properties of PCBs are weak [50], whereas some PCB mixtures in other systems exhibit antiestrogenic activity [51]. However, it is possible that even weak estrogenic exposure during early development may have a profound impact upon postnatal health.

Therefore studies in the 90s [52,53] suggested the hypothesis that human exposure to environmental levels of organochlorines—including PCBs—would favor an estrogenic overactivity leading to an increase in estrogen-dependent formation of mammary or endometrial tumors. Nevertheless this notion was not supported by the existing in vitro, animal and epidemiological evidence at that time but it was considered not conclusively rejected on the basis of the few available data. Two case-control studies [26,54] where the serum concentrations of PCBs in sick and healthy patients was compared, resulted in a no significant association of increasing levels of PCB exposure with endometrial cancer risk.

On the other hand, Yoshikawa et al. [55] performed an in vivo study where female adult Harlan Sprague-Dawley rats were exposed for 14, 31 and 53 weeks or for two years to different EDCs including PCBs (3,3’,4,4’,5 pentachlorobiphenyl (PCB 126); 2,3,4,7,8-pentachlorodibenzofuran (PeCDF); 2,2’,4,4’,5,5’-hexachlorobiphenyl (PCB 153); 2,3’,4,4’,5-pentachlorobiphenyl (PCB 118) a binary mixture of PCB 126 and 153; or a binary mixture of PCB 126 and PCB 118) and resulted in an increasing of uterine squamous cell carcinoma uterine squamous cell carcinoma in the 300 ng/300 μg/kg core group that received the binary mixture of PCB 126 and 153 and in a clearly increasing incidence of uterine carcinoma in the 1000 and 4600 μg/kg PCB 118 core group and the 4600 μg/kg stop group.

In the studies of PCB 126, the tertiary mixture, and the binary mixture of PCB 126 and PCB 118, no increased incidence of any change occurred in the reproductive systems. The range of changes seen with the different compounds suggested that more than one mechanism may be involved in promoting the female reproductive pathology.

Actually PCBs dioxin-like are known to interact with the aryl hydrocarbon receptor (AhR); endometrial cancer cells are both Ah- and E2- responsive and express the AhR and ER and in cell proliferation and transcriptional activation studies, and several different AhR agonists inhibit E2-mediated responses. These results would demonstrate inhibitory crosstalk between the AhR and ER signalling pathways in endometrial cells [43] and would explain these controversial results.

## 3. BPA and Endometrial Cancer

Bisphenol A (BPA) is one of the most widely produced chemicals worldwide and is often used in the production of food and beverage containers. As a result of BPA contact with food, drink and toiletries, its ingestion and absorption by humans has been growing. The tolerable daily intake (TDI) of 50 μg/kg bw/day in the USA and 10 μg/kg bw/day in the European Union is considered to be higher than the daily human uptake of BPA (<1 μg/kg bw/day). However, this setting did not take into account the vulnerability to BPA exposure during prenatal and neonatal development [58].

As a matter of factsin an vivo animal study where outbred female CD-1 mice were treated on days 1–5 with subcutaneous injections of BPA (10, 100 or 1000 μg/kg/day), and examined at 18 months, an increase in cystic ovaries and cystic endometrial hyperplasia (CEH) was demonstrated in the BPA-100 group as compared to controls, suggesting that BPA causes long-term adverse effects if exposure occurs during critical periods of differentiation [59]. Furthermore, many studies have demonstrated that dosages below the current TDI cause significant effects in animal models [60].

Many studies have highlighted the correlation between the increase of BPA level in the environment and the incidence of tumor in humans. In human carcinogenesis, the overexpression of cyclooxygenase-2 (COX-2) and epithelial-mesenchymal transition (EMT) are closely related with tumor development and it had been demonstrated that BPA can induce EMT and COX-2 expression in human endometrial carcinoma cells line (RL95-2): BPA increased growth rate and colony-forming efficiency in a dose-dependent manner, induced EMT and COX-2 gene expression and promoted the migration and invasion ability of RL95-2 cells [61] (Table 3).

In a study where the ability of BPA to affect human estrogen receptor (ER) binding, expression of progesterone receptor (PR) mRNA and protein, and cell proliferation in the human endometrial cell line was evaluated, although less potent than 17β-estradiol, BPA was able to bind to the human uterine ER. BPA also induced both mRNA and protein to levels similar to E2, suggesting an ER-mediated pathway [62].

BPA is able to induce thousands of estrogen receptor1 (ESR1) binding sites and change the expression of a subset of genes (more often up-regulated) affected by E2 [63]. Furthermore it has been shown to activate MAPK1 (also known as ERK) kinase [64], AKT1 (also known as AKT) kinase, and PIK3CA (also known as PI 3-kinase) [65] in different cell types.

Although BPA is identified as an estrogenic substance and may activate both ERα and ERβ, this activation would be both cell-type- and concentration- dependent [66]. Furthermore it also weakly activates the IGF signalling pathway via ERα in the uterus of ovx adult mice, leading to an increase in mitotic cells, indicating that BPA may also promote epithelial proliferation via others alternative signalling pathways [67].

Furthermore, in addition to the capacity to bind to nuclear estrogen receptors (ERs) α- and β- [68,69], other mechanisms of action can also result from binding to other targets within the nucleus or in the cell membrane [70]. In a series of in vitro assays, Li et al. [71,72] demonstrated that BPA and the fluorinated derivative bisphenol AF (BPAF) may activate both ERα and ERβ but that activation was both cell-type- and concentration-dependent. For example, in Ishikawa cells (endometrial adenocarcinoma cell line), BPA at concentrations lower than 10  nM antagonised E2-mediated ERα activation of luciferase activity while in HeLa cells (cervical adenocarcinoma cell line) similar concentrations of BPAF antagonised E2-mediated ERβ activation of luciferase activity [72]. BPA and BPAF at concentrations greater than 10  nM and up to 100  nM were reported to act as agonists through both ERα and ERβ [71,72].

Notwithstanding, serum concentrations of BPA measured in both pre- and postmenopausal women were significantly lower in patients with complex endometrial hyperplasia or endometrial cancer than in healthy controls [73]. These findings suggest the presence of associations between BPA exposure and complex endometrial hyperplasia and endometrial cancer, but without a linear dose-response curve. The mode of action of BPA may be more complex than expected and the contradictory results address the complex mechanisms of linkage between occurrence of estrogen-dependent diseases and endocrine disruption.

BPA has structural similarities to E2 and is considered mainly as an ERα and ERβ agonist [60] but it can also affect other endocrine pathways, e.g., by acting as antagonist of the androgen receptor (AR) or as agonist of the aryl hydrocarbon receptor (AhR), involved in cross talk processes with ERs, AR and other nuclear receptors (NRs), and of pregnane X receptor (PXR) [74,75,76]. Furthermore, the nuclear estrogen-related receptor γ is also activated by BPA and interacts with the ligand domain of ERs [74,77]. Other targets include non-classical membrane estrogen receptors (mERs), such as the G-protein-coupled receptor 30 (GPR30) [78,79].

In an in vitro study [25], using a yeast strain incorporating a vitellogenin A2 ERE-LacZ reporter gene into the genome, it was found that BPA induced expression of the reporter in colonies transformed with the ERα expression plasmid, illustrating BPA-mediated regulation within a chromatin context. Additionally, a reporter gene transiently transfected into the endometrial cancer (Ishikawa) cell line also showed BPA activity, although at 100-fold less potency than E2. It is relevant that a number of growth- and development-related genes, such as HOXC1 and C6, Wnt5A, Frizzled, TGFbeta-2, and STAT inhibitor 2, were found to be affected exclusively by BPA.

In a study investigating the effect of BPA on human endometrial stromal cell (ESC) differentiation, BPA was found to decrease proliferation of ESC and decrease expression of mRNAs encoding CYP11A1, HSD17B1 and HSD17B2; however, this effect was only observed with high (50–100 μM) doses [80].

Aside from the receptor mediated mechanism, BPA exerts its action through epigenetic mutations, altering developmental pathways and cell processes [81,82]. In fact, DNA methylation analysis showed that the BPA exposure induced the hypermethylation of BCL2L11, PARD6G, FOXP1 and SFRS11, as well as the hypomethylation of NUP98 and CtIP (RBBP8). This indicated that normal cells exposed to BPA have increased expressions of genes involved in DNA repair in order to overcome the DNA damage induced by this chemical suggesting that mutation carrier patients could be more susceptible to the cancerogenic effects of BPA [83].

## 4. Dioxins and Endometrial Cancer

The term dioxins is used to specify polychlorinated dibenzo-*p*-dioxins (PCDD), dibenzofurans (F) and dioxin-like polychlorinated biphenyls (PCBs). Many of the congeners are proved to be highly toxic, but 2,3,7,8-tetrachlorodibenzo-*p*-dioxin (TCDD) is the most toxic congener, and probably the most toxic compound ever synthesized by man, being widespread and highly persistent in the environment, and accumulating in living organisms, especially in fat tissue. The dioxins are known to adversely affect the reproductive system in part due to their ability to alter hormone levels in many species [84,85].

TCDD and related compounds induce a broad spectrum of biochemical and toxic responses and disrupt multiple endocrine pathways.Reproductive cancer risks were first reported following the “Seveso Accident” in 1976, which caused exposure to TCDD of a large residential population at a chemical plant in Seveso, Italy [86,87,88].Nevertheless in the case-control study, where the Seveso population accidentally exposed to TCDD were followed up for cancer occurrence in 1977–1986, there was no correlation between TCDD exposure and endometrial cancer, and as a matter of fact, TCDD was listed as a Group 1 carcinogen (nongenotoxic, carcinogenic to humans) by the International Agency for Research on Cancer in 1997 [89].

Although the exact mechanism of TCDD-induced toxicity is not completely understood, many studies indicate that the most of TCDD effects are mediated through the aryl hydrocarbon receptor (AhR), a ligand-activated member of the bHLH-PAS family of transcription factors [90,91,92]. The AHR typically resides quiescently as a cytoplasmic complex with its chaperone proteins heat shock protein 90 (HSP90) and AHR-interacting protein (AIP). Upon ligand binding, the AHR translocates to the nucleus where it disaggregates from the chaperone proteins and heterodimerizes with the AHR nuclear translocator (ARNT) [93,94]. The resulting complex binds to aryl hydrocarbon response elements (AHREs) in DNA and alters the transcription of target genes such as Cyp1a1 [94,95].

Laboratory research has focused on characterizing aryl hydrocarbon receptor (AhR)-mediated antiestrogenicity in the rodent uterus and mammary and in human breast cancer cells [96]. TCDD was demonstrated to inhibit multiple estrogen (E2)-induced responses in these tissues including development or growth of human mammary and endometrial cancer cells, carcinogen-induced mammary cancer in rats, and mammary cancer in mice bearing breast cancer cell xenografts [27].

Indeed TCDD has been reported to cause anticarcinogenic effects in hormone-dependent tissues such as mammary, uterus and the pituitary of female rats fed the compound for a period of two years [97]. Several in vivo studies in rats have also suggested that TCDD exposure counteracts the effects of estrogen with regard to uterine hypertrophy, peroxidase activity, and binding activities of estradiol receptor (ER), progesterone receptor (PR) and epidermal growth factor receptor [98]. Nevertheless it has to be considered that TCDD, does not persist in rats [99], on the contrary of what does in humans.

Furthermore in ER-positive human breast cancer cell lines, TCDD inhibits 17β-estradiol(E2)-dependent proliferation [97], secretion of tissue plasminogen activator [100], postconfluent focus formation [101,102], and secretions of E2-induced proteins like cathepsin-D or pS2 [97] but none of these effects has been found in ER-negative breast cancer [103].

This is a relevant evidence because demonstrate that TCDD does not interact directly with ER or PR and therefore, the antiestrogenic effects cannot be explained by direct interaction of TCDD with those receptors [104]. Rather, antiestrogenic activity might be explained by a decrease in the amount of ER [105,106] or inhibition of estradiol-induced gene transcription by transcriptional interference with the liganded AhR- ARNT complex [107] with XRE elements found to be present in E2-inducible genes [108].

Although these studies indicate that dioxins may be protective against estrogenic stimulation and development of hormonal dependent cancer, many in vitro studies demonstrate other mechanisms which would correlate TCDD to endometrial cancer. The exposure to TCDD also modulates the immune response by influencing the production and action of endometrial cytokines and chemokines, destroying mucosal immunity of the reproductive tract and re-directing the tissue distribution and behavior of leukocytes [109]. Over the past two decades, our understanding of inflammation in tumorigenesis [110] has helped elucidate this further mechanism which would be implicated in the correlation between dioxin and endometrial cancer.

Charles et al. [111] investigated the potential role of TCDD in uterine growth through a human endometrial adenocarcinoma cell line (RL95-2). Western immunoblot analysis showed a maximal induction of cytochrome P4501A1 (CYP1A1) at 1 nM TCDD. Furthermore TCCD significantly increased mRNA levels for interleukin-1beta (IL-1beta) at 6 h, and for urokinase plasminogen activator (uPA) and tumor necrosis factor-alpha (TNF-alpha) at 36 h.

Jana et al. [112] investigated the mechanism of the response of human uterine endometrial carcinoma cells, RL95-2 (epithelial carcinoma cells of the uterus) and KLE (adenocarcinoma cells of the uterus), to 2,3,7,8-tetrachlorodibenzo-*p*-dioxin (TCDD). RL95-2 cells were highly responsive to TCDD in terms of cytochrome P4501A1 (CYP1A1), cytochrome P4501B1 (CYP1B1), and plasminogen activator inhibitor-2 (PAI-2), whereas KLE cells showed little stimulatory effects only at high doses.

Of relevance had been the study of Yoshizawa et al. [55] where female adult Harlan Sprague-Dawley rats were exposed for 14, 31 and 53 weeks or for two years to different EDCs including 2,3,7,8-tetrachlorodibenzo-*p*-dioxin (TCDD) resulting in a marginally or significantly increasing of uterine squamous cell carcinoma, respectively, in the 6 ng/kg core and 100 ng/kg stop-exposure groups (Table 4).

Therefore TCDD would induce several AhR-mediated changes in gene expression an tissue-/species-specific toxicities and is a potent inhibitor of estrogen-mediated activity: both tumorigenic and anticarcinogenic responses occur, including inhibition of estrogen-dependent uterine and mammary tumor formation and and growth, via inhibitory AhR–estrogen receptor cross-talk [27,113]. TCDD and other AhR ligands suppress estradiol-induced responsesin the rodent uterus, mammary tumors, and human breast cancer cells [114]. The increase of the uterine squamous cell carcinoma risk would be due to the fact that TCDD disrupts retinoid homeostasis and causes vitamin A deficiency [115,116,117] in the systemic organs [114,115,116,117,118].

Hepatic vitamin A is reduced in rats following dietary exposure to TCDD for thirteen weeks [117,121,122] and characteristic of retinoid deficiency is abnormal epithelial differentiation to a keratinized squamous phenotype [123,124,125,126,127].

The action of dioxin-like compounds (DLCs) might, therefore, be also due to a disruption of retinoid action, leading to altered growth and differentiation of the endometrial epithelium resulting in squamous metaplasia (SM) and squamous cell carcinoma (SCC).

On this point TCDD and DLCs are documented to induce epithelial SM and SCC in other organs, including gingival squamous-cell hyperplasia and/or SCC, SM, and/or SCC in the lung, and squamous hyperplasia in the forestomach [128,129].

At last but not least, a possible association between dioxin and endometriosis [130] has been reported and adenomyosis is known to be associated with a 4- to 5-fold increased risk of endometrial cancer [131]. Postoperative pathological examinations of endometrial cancer patients frequently reveal the coexistence of uterine adenomyosis. However, the influences of uterine adenomyosis on the prognosis of endometrial cancer are still unclear. There were reports of muscle invasion being facilitated by the presence of uterine adenomyosis in endometrial cancer patients [132,133] which showed that prognoses are better in cases with uterine adenomyosis than in those without [134,135]. However, the number of cases supporting these claims is small, and therefore, such claims remain inconclusive.

According to a number of histopathological studies endometriosis-associated ovarian cancer (EAOC) may arise from atypical endometriosis of the ovary and this heterogeneous condition is histologically characterized by hyperplasia of endometrial glands with cytological atypia or presence of atypical hobnail cells within ovarian endometriosis [136]. Thus, in the adenomyosis-associated endometrial cancer might be involved the same atypical cells transformation.

## 5. Cadmium and Endometrial Cancer

Cadmium (Cd) belongs to the heavy metals, and is very common both in the industry and in the natural environment. It is classified as the 7th substance on the Agency for Toxic Substances and Disease Registry Priority List of Hazardous Substances [137].

The main source of exposure to cadmium for the general population is smoking tobacco or consumption of food containing cadmium [138,139]. Inhalation absorption is also efficient and thus smokers have 4–5 times greater blood cadmium levels than non-smokers [137,140]. Women are thought to be at greater risk of increased cadmium accumulation, as the concentrations of cadmium in blood and urine of females are significantly higher than in males due to the lower levels of iron [141]. Cd has a half-life of 3–4 months in blood and then it is transported to other parts of the body. It accumulates mainly in kidneys, liver and lungs [142]. In addition, as our earlier studies point out, it is also retained in reproductive organs [143]. Cadmium elimination from the organism is extremely slow and its biological half-life is 10–30 years [144]. Recent studies have investigated the effect of cadmium exposure in the general population and suggested that cadmium may have adverse effects at lower exposure levels than previously expected [138].

Due to the endocrine-disrupting properties of cadmium [145], it has been suggested that environmental exposure to it may cause estrogen-dependent diseases such as breast cancer and endometrial cancer [146,147].

Multiple mechanisms potentially link cadmium to cancer, including oxidative stress and inflammation [148,149], interference with DNA repair [150,151], and alterations of DNA methylation [152]. More relevant to hormone-related cancers, perhaps, is evidence that cadmiummay act on estrogenic signaling pathways [153], resulting in proliferation of breast cancer cells in vitro [154], and inducing uterus and mammary gland weight increase in rats [155]. Long-term treatment with low concentrations of cadmium can malignantly transform breast cells in vitro, and the oncogenic action would be independent of estrogen receptor-α [156] (Table 5).

Although the cadmium concentration in cancerous endometrial samples might not be different than that found in noncancerous tissue [160] or only slightly lower than that in non-lesion uterine tissue [143], there is evidence that cadmium could be a risk factor for breast, endometrial or ovarian cancers in postmenopausal women. The association between dietary cadmium intake and risk of these cancers has been demonstrated in the large, well-characterized Women's Health Initiative (WHI). Analyzing 155,069 women little evidence that dietary cadmium is a risk factor for breast, endometrial, or ovarian cancers in postmenopausal women was found [161].

Prospective studies in Sweden showed an association between estimated dietary cadmium and endometrial cancer [146] and postmenopausal breast cancer [162], but not ovarian cancer [163]. In contrast, similar studies from the United States [161] and Japan [159] did not observe an association of dietary cadmium with postmenopausal breast cancer risk or risk of any cancer, respectively.

Cho et al. [159] conducted a meta-analysis which combined and analyzed the results of previous studies that have investigated the association of dietary cadmium intake and cancer risk. The analysis found a positive association between dietary cadmium intake and cancer risk among studies conducted in Western countries, particularly with hormone-related cancers such as the endometrial one.

Of interest is the association between cadmium and endometrial cancer depending on the BMI: Eriksen et al. estimated dietary cadmium intake in the 1993–1997 Diet, Cancer and Health cohort at enrollment. A positive association between cadmium and endometrial cancer for the women with BMI < 25 was found, whereas an inverse association was seen for the women with BMI ≥ 25 [157].

Several studies found that stronger associations between cadmium and cancer risk were observed among individuals with low bioavailable estrogen such as low body mass index (BMI) [162,163,164,165] or nonusers of postmenopausal hormones [146].

## 6. Conclusions

The environment has a large biological impact on human health and disease. During decades, the long-term and persistent effects of chemical compounds to which humans are exposed daily have been ignored because of the lack of scientific information and public awareness. The carcinogenic effects of EDCs are hard to define for the following reasons:

1. Difficulty in the evaluation of the lifelong exposure (e.g., non persistent EDs like most pesticides, phtalates and BPA do not cause a body burden, thus, measuring the level of the substance may not reflect the possible relationship between exposure and slow-onset of the diseases such as cancer);
2. Lipophilic EDCs are stored in the fat tissue with a bio-accumulation of minimal daily doses, therefore its rapid mobilization during a drastic diet can expose the person to high doses of the chemical which are not well evaluated in the studies;
3. EDCs can have effects at low doses that are not predicted by effects at higher doses (it cannot be assumed that there is a threshold because hormones can regulate the hormone receptors expression resulting in an inverted U dose-response curve); NMDR can arise from numerous molecular mechanisms such as opposing effects induced by multiple receptors differing by their affinity, receptor desensitization, negative feedback with increasing dose, or dose-dependent metabolism modulation;
4. Individuals can be concurrently exposed to different EDCs (cocktail effect) generating unpredictable interactions, because the epigenetic effects can also affect future generations especially if the exposure had been in vulnerable developmental periods (e.g., pre-natal and pubertal periods); EDCs are associated with declining human reproductive health, as well as an increasing incidence of cancers of the reproductive system. Verifying such links requires animal models exposed to “real-life”, environmentally relevant concentrations/mixtures of EDC, particularly in utero, when sensitivity to EDC exposure is maximal;
5. Some individuals can have a particular susceptibility to ED carcinogenesis;
6. It’s not always evaluated that EDCs can also have indirect effects on carcinogen metabolism, immune system, oxidation and inflammation;
7. The competing interests may limit research and public information on ED effects.

Generally exposures at critical windows of early development (fetal, birth, puberty) have the most dramatic impact on later life disease development or abnormal physiology. This developmental concept is referred to as the developmental origins of health and disease [166]. Since epigenetics and genetics cooperate in regulating genome activity (gene expression), a cascade of genetic and epigenetic events are required to achieve normal adult development (differentiation) [167].

In conclusion, the carcinogenic effects of endocrine disruptors is plausible although additional studies are needed to clarify their mechanisms and entities. Nevertheless, to reduce ED exposure is mandatory to implement necessary measures to limit exposure, particularly during those periods of life most vulnerable to the impact of oncogenic environmental causes, such as embryonic period and puberty.

## Figures and Tables

**Table 1:** Articles on EDCs and endometrial cancer published until 2016.

Type of Article	Main Author (Year)	Subject
Review	Klinge (2015) [18]	miRNAs regulated by estrogens, tamoxifen, and endocrine disruptors and their downstream gene targets.
Review	Gibson (2015) [9]	Endocrine disruption of oestrogen action and female reproductive tract cancers.
Review	Rochester (2015) [19]	Bisphenol A and human health: A review of the literature
In vitro study	Nordeen (2013) [20]	Endocrine disrupting activities of the flavonoid nutraceuticals luteolin and quercetin
In vitro study	Kortenkamp (2011) [21]	Are cadmium and other heavy metal compounds acting as endocrine disrupters?
In vitro study	Boehme (2009) [22]	Gene expression profiling in Ishikawa cells: a fingerprint for estrogen active compounds
In vitro study	Xu (2008) [23]	Development of a stable dual cell-line GFP expression system to study estrogenic endocrine disruptors.
Review	Caserta (2008) [24]	Impact of endocrine disruptor chemicals in gynaecology.
In vitro study	Singleton (2006) [25]	Gene expression profiling reveals novel regulation by bisphenol-A in estrogen receptor-alpha-positive human cells.
Case Control Study	Hardell (2004) [26]	Adipose tissue concentrations of p,p’-DDE and the risk for endometrial cancer.
In vitro study	Safe (1998) [27]	Ah receptor agonists as endocrine disruptors: antiestrogenic activity and mechanisms.
In vitro study	Garey (1998) [28]	Estrogenic and antiprogestagenic activities of pyrethroid insecticides.

**Table 2:** Polychlorinated biphenyls (PCB) and endometrial cancer: mechanisms of action and correlated published studies.

Chemical(s)	Pathways of Exposure	Mechanism of Action	Authors (Year)	Results
Polychlorinated biphenyls (PCBs)	Food chain (fat-rich food, e.g., milk and derivates, fatty fish), living environment	Alteration steroid hormone metabolism/transport, ability to bind with the tyroxin transport protein transthyretin (TTR), interaction with thyroid hormone receptors, neuroendocrine effects. PCBs dioxin-like: Aril hydrocarbon Receptor interaction leading to altered steroid hormone metabolism and neuroendocrine effects including on thyroid	Chen, et al. 2015 [45]	It was observed that PCBs affected the expression of inflammatory factors through ER and AHR receptors but no toxic effects were observed on estrogen metabolism.
			Reich, et al. 2010 [56]	Case Control Study where high levels of PCB and others EDCs where found in the abdominal adipose tissue of two cases of endometrial stromal sarcoma
			Yoshizawa, K. et al. 2009 [55]	In vivo study where female adult Harlan Sprague-Dawley rats were exposed for 14, 31 or 53 weeks or for two years to different EDCs including PCB126, PeCDF, PCB153, PCB118, a binary mixture of PCB126 and 153; or a binary mixture of PCB126 and PCB118; and resulted in an increasing of uterine squamous cell carcinoma uterine squamous cell carcinoma in the 300 ng/300 μg/kg core group that received the binary mixture of PCB126 and 153 and in a clearly increasing incidence of uterine carcinoma in the 1000 and 4600 μg/kg PCB118 core group and the 4600 μg/kg stop group. In the studies of PCB 126, the tertiary mixture, and the binary mixture of PCB126 and PCB118, no increased incidence of any change occurred in the reproductive systems. The range of changes seen with the different compounds suggests that more than one mechanism may have been involved in promoting the female reproductive pathology.
			Hardell, L. et al. 2004 [26]	Case control study where it was analyzed the adipose tissue concentration of HCB, p,p’-DDE, chlordanes and polybrominated biphenyls in 76 cases with endometrial cancer and 39 controls with benign endometrial hyperplasia suggesting an interaction between p,p’-DDE and estrogen replacement drugs in the etiology of endometrial cancer, although no significant associations were found.
			Weiderpass, E. 2000 [57]	Case Control study where was measured serum concentrations of 10 chlorinated pesticides and 10 PCB congeners in 154 endometrial cancer and 205 population controls and resulted a no significant associations of increasing levels of pesticide or PCB exposure with endometrial cancer risk.
			Sturgeon, S.R. 1998 [54]	Multicenter case-control study: the findings did not support the hypothesis that organochlorine compounds are linked to the development of endometrial cancer.
			Adami 1995 [53]	Review that summarizes the evidence regarding whether certain organochlorine compounds increase the risk of breast and endometrial cancers through their estrogenic potential and resulted that no analytic epidemiologic studies of endometrial cancer were published at that data.
			Ahlborg 1995 [52]	Review that summarizes the evidence regarding whether certain organochlorine compounds increase the risk of breast and endometrial cancers through their estrogenic potential and resulted that the hypothesis that human exposure to environmental levels or organochlorines would favor an estrogenic overactivity leading to an increase in estrogen-dependent formation of mammary or endometrial tumors is not supported by the existing in vitro, animal and epidemiological evidence.

**Table 3:** Bisphenol A (BPA) and endometrial cancer: mechanisms of action and correlated published studies.

Chemical	Pathways of Exposure	Mechanism of Action	Authors (Year)	Results
Bisphenol A (BPA)	Food chain (e.g., plastics in contact with food), consumer products (e.g., dental sealant, plastic additive, etc.)	Estrogen agonists-ER alpha, epigenetic mutations	Wang, K.H. et al. 2015 [61]	The results show that BPA increased growth rate and colony-forming efficiency in a dose-dependent manner, induced EMT and COX-2 gene expression and promoted the migration and invasion ability of RL95-2 cells.
			Gibson, D.A. et al. 2014 [9]	Review that summarizes how BPA is identified as an estrogenic substance and may activate both ERα and ERβ but that activation would be both cell-type- and concentration-dependent.
			Rochester, J.R. et al. 2013 [19]	Review shows the associations between BPA exposure and adverse perinatal, childhood, and adult health outcomes, including reproductive and developmental effects, metabolic disease, and other health effects.
			Gertz, et al. 2012 [63]	In vitro study where it was demonstrated that BPA and genistein induce thousands of estrogen receptor1 (ESR1) binding sites and change the expression of a subset of genes (more often up-regulated) affected by E2, representing 26% and 6% respectively.
			Boehme, et al. 2009 [22]	It was showed a divergent gene expression patterns of the phytoestrogens, as well as weaker estrogenic gene expression regulation determined for the anthropogenous chemicals BPA and o,p’-DDT.
			Newbold, R.R. 2007 [59]	There was a statistically significant increase in cystic ovaries and cystic endometrial hyperplasia (CEH) in the BPA-100 group as compared to Controls, suggesting that BPA causes long-term adverse effects if exposure occurs during critical periods of differentiation.
			Singleton, D.W. et al. 2006 [25]	It has been relevant how a number of growth- and development-related genes, such as HOXC1 and C6, Wnt5A, Frizzled, TGFbeta-2, and STAT inhibitor 2, were found to be affected exclusively by BPA.
			Hiroi, H. 2004 et al. [73]	Human in vivo study suggests the presence of associations between BPA exposure and complex endometrial hyperplasia and endometrial cancer.
			Kurosawa, T. et al. 2002 [66]	In vitro study where was performed a luciferase assay on three independent cell lines derived from different tissues transfected with either human ERα cDNA or ERbeta cDNA, indicating that BPA only acts as an agonist of estrogen via ERbeta whereas it has dual actions as an agonist and antagonist in some types of cells via ERα. Thus, the activity of BPA may depend on the ER subtype and the tissue involved.
			Bergeron, et al. 1999 [62]	BPA was able to bind to the human uterine ER and to induce both mRNA and protein to levels similar to E2.

**Table 4:** Dioxins and endometrial cancer: mechanisms of action and correlated published studies.

Chemical	Pathways of Exposure	Mechanism of Action	Authors (Year)	Results
Dioxins	Food chain (fat-rich food, e.g., milk and derivates, fatty fish), living environment	Aril hydrocarbon Receptor interaction leading to altered steroid hormone metabolism and neuroendocrine effects including on thyroid	Yoshizawa, K. et al. 2009 [55]	In vitro study where female adult Harlan Sprague-Dawley rats were exposed for 14, 31 or 53 weeks or for two years to different EDCs including 2,3,7,8-tetrachlorodibenzo-*p*-dioxin (TCDD) resulting in a marginally or significantly increasing of uterine squamous cell carcinoma rispectively in the 6 ng/kg core and 100 ng/kg stop-exposure groups.
			Jana, N.R. et al. 1999 [112]	In vitro study where it was investigated the mechanism of the response of human uterine endometrial carcinoma cells, RL95-2 (epithelial carcinoma cells of the uterus) and KLE (adenocarcinoma cells of the uterus), to 2,3,7,8-tetrachlorodibenzo-*p*-dioxin (TCDD). RL95-2 cells were highly responsive to TCDD in terms of cytochrome P4501A1 (CYP1A1), cytochrome P4501B1 (CYP1B1), and plasminogen activator inhibitor-2 (PAI-2), whereas KLE cells showed little stimulatory effects only at high doses.
			Ricci, M.S. et al. 1999 [119]	In vitro study where it was demonstrated that TCDD exerts its toxic action via the aryl hydrocarbon (Ah) receptor, which induces a battery of xenobiotic-metabolizing enzymes, including the cytochrome P450 isozyme, CYP1A1. TCDD-induced 7-ethoxycoumarin-O-deethylase activity was reduced 75% in cultured human endometrial ECC-1 cells exposed to various concentrations of 17beta-estradiol for up to 72 h, with a half-maximal effective concentration (EC50) of 0.9 nM.
			Charles, G.D. et al. 1997 [111]	In vitro study where it was investigate the potential role of TCDD in uterine growth utilizing a human endometrial adenocarcinoma cell line (RL95-2). Western immunoblot analysis showed a maximal induction of cytochrome P4501A1 (CYP1A1) at 1 nM TCDD. Furtherome TCCD significantly increased mRNA levels for interleukin-1beta (IL-1beta) by 6 h, and for urokinase plasminogen activator (uPA) and tumor necrosis factor-alpha (TNF-alpha) by 36 h.
			Bertazzi, A. et al. 1993 [120]	Case control study where Seveso Population accidentally exposed to TCDD were followed up for cancer occurrence in 1977–1986. No cases of endometrial cancer was detected.

**Table 5:** Cadmium and endometrial cancer: mechanisms of action and correlated published studies.

Chemical	Pathways of Exposure	Mechanism of Action	Authors (Year)	Results
Cadmium	Food Chain (e.g., refined food as flour, rice, sugar; seafood), cigarette smoking	Estrogen agonist- ER alpha	Eriksen, K.T. et al. 2014 [157]	It was found a positive association between cadmium and endometrial cancer for the women with BMI < 25, whereas an inverse association was seen for the women with BMI ≥ 25.
			Adams, S.V. et al. 2014 [158]	Case control study where it was examined the association between dietary cadmium intake and risk of these cancers in the large: it was found little evidence that dietary cadmium is a risk factor for breast, endometrial, or ovarian cancers in postmenopausal women.
			Cho, Y.A. et al. 2013 [159]	The analysis found a positive association between dietary cadmium intake and cancer risk among studies conducted in Western countries, particularly with hormone-related cancers such as the endometrial one.
			Akesson, A. et al. 2008 [146]	The results dimonstrated that the Cadmium intake was statistically significantly associated with increased risk of endometrial cancer in all women
			Yaman, M. et al. 2007 [160]	The amount of Cadmium found in cancerous endometrial samples were not found to be different than those in noncancerous tissues.
			Nasiadek, M. et al. 2005 [143]	In the investigated tissues, the correlation between Cd concentration and age was found, but no effect of menopausal status or smoking habits on Cd level was detected.
